# From Supramolecular Hydrogels to Multifunctional Carriers for Biologically Active Substances

**DOI:** 10.3390/ijms22147402

**Published:** 2021-07-09

**Authors:** Joanna Skopinska-Wisniewska, Silvia De la Flor, Justyna Kozlowska

**Affiliations:** 1 Faculty of Chemistry, Nicolaus Copernicus University in Torun, Gagarin 7, 87-100 Torun, Poland; joanna@umk.pl; 2 Department of Mechanical Engineering, Universitat Rovira i Virgili, Av. Països Catalans 26, 43007 Tarragona, Spain; silvia.delaflor@urv.cat

**Keywords:** supramolecular hydrogels, non-covalent interactions, drug delivery, controlled release

## Abstract

Supramolecular hydrogels are 3D, elastic, water-swelled materials that are held together by reversible, non-covalent interactions, such as hydrogen bonds, hydrophobic, ionic, host–guest interactions, and metal–ligand coordination. These interactions determine the hydrogels’ unique properties: mechanical strength; stretchability; injectability; ability to self-heal; shear-thinning; and sensitivity to stimuli, e.g., pH, temperature, the presence of ions, and other chemical substances. For this reason, supramolecular hydrogels have attracted considerable attention as carriers for active substance delivery systems. In this paper, we focused on the various types of non-covalent interactions. The hydrogen bonds, hydrophobic, ionic, coordination, and host–guest interactions between hydrogel components have been described. We also provided an overview of the recent studies on supramolecular hydrogel applications, such as cancer therapy, anti-inflammatory gels, antimicrobial activity, controlled gene drug delivery, and tissue engineering.

## 1. Introduction

Hydrogels are a wide group of amazing materials that can increase in size due to their ability to absorb a large volume of water or other fluids, while maintaining integrity. Their mechanical and swelling properties may often change depending on environmental factors. These properties make hydrogels willingly used in various fields, e.g., food processing, agriculture, adhesives, personal care products, and, most of all, the biomedical field (from drug delivery systems and wound healing, to cell immobilization and tissue engineering [1,2]). The unique physical properties of hydrogels make them very useful as carriers of drugs and biologically active substances. High water content and flexibility make hydrogels similar to biological tissues. They are biocompatible and absorb proteins from body fluids to a minimal degree. Their porosity can be easily regulated by the cross-linking degree, and molecules of different sizes may be loaded into the hydrogel structure. The active substances released from hydrogel may also be modified by diffusion and swelling control, as well as by chemical processes, such as hydrolytic or enzymatic cleavage of the hydrogel network. A special advantage of the hydrogels are their ability to respond to various stimuli, such as pH or temperature changes, magnetic/electric field, light, and the presence of ions or other chemical molecules [3,4,5,6,7].

Generally, hydrogels are classified into two types: chemical and physical hydrogels. The first group is formed by the permanent covalent cross-linking of hydrophilic, natural or synthetic, polymers. Such hydrogels are relatively stable and resistant to degradation, but also show low transparency and often are brittle. When their covalent cross-links are broken, the 3D structure is irreversibly destroyed. This type of hydrogel does not possess a self-healing ability. Incorporation of drugs into chemical hydrogels may be realized by sorption into the previously prepared material. This process is time-consuming, and loading content is limited. Drugs may be also loaded before hydrogel cross-linking. However, the cross-linking reaction may cause drugs’ permanent conjugation to the polymer matrix, and the hydrogel may become non-biodegradable due to its composition modification. Chemical structure and pharmacokinetics of drugs can be affected too [8,9].

The hydrogels of the second group, also called supramolecular hydrogels, are formed via reversible, non-covalent interactions between macromolecules and low-molecular-weight gelators too [3,8]. Generally, the formation of this kind of hydrogel occurs in two steps: self-assembly and cross-linking. The structure is stabilized by the creation of, for example, hydrogen bonds, hydrophobic, electrostatic, coordination, and host–guest interactions [8]. The gel formation process can be initiated by a variety of physical (e.g., temperature, ultrasound, light, magnetic field) and chemical factors (e.g., pH changes), as well as enzymatic reactions [10]. Supramolecular hydrogels, the same as chemical ones, show moderate mechanical properties. Their great advantage is their ability to self-repair damage and return to their original properties (self-healing properties) as a result of the creation of reversible non-covalent interactions. Usually, they are biocompatible and easily degradable. Moreover, the supramolecular hydrogels show a reversible sol–gel transition as a bio-related stimuli response. This makes them useful to prepare, e.g., injectable hydrogels. These properties provide the opportunity for the precise delivery of active substances [1,2,7,8,11,12,13].

Our review consists of two main parts. In the beginning, the most important types of interactions occurring in supramolecular hydrogels are discussed. The hydrogen bonds, hydrophobic interactions, ionic interactions, metal–ligand coordination, host–guest interactions, and systems where these interactions play a key role are described. Then, we present the latest developments on the use of supramolecular gels in cancer therapy and of anti-inflammatory and antimicrobial hydrogels for controlled gene drug delivery and applied in tissue engineering. Finally, we highlight the future challenges that await scientists working in this field.

## 2. Interactions in Supramolecular Hydrogels

### 2.1. Hydrogen Bonds

**Figure 1:**
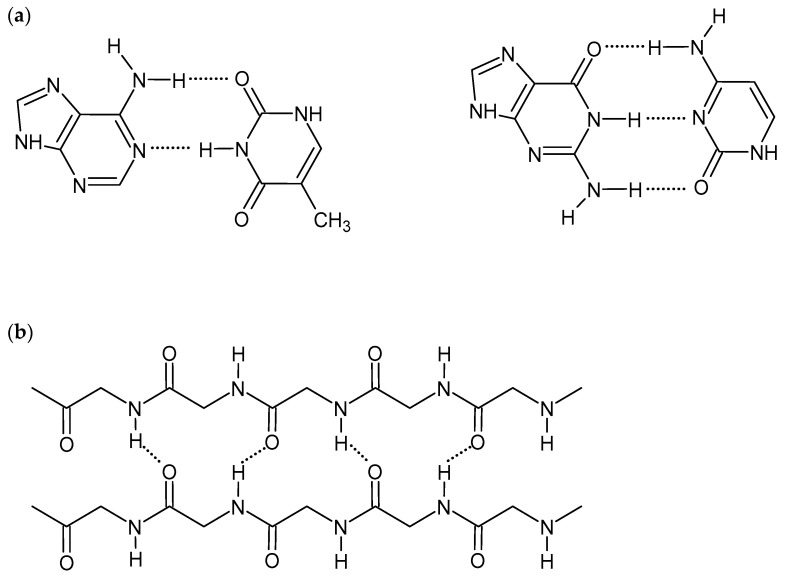
Scheme of hydrogen bonds between: (**a**) DNA base pairs, (**b**) proteins β-sheet structure.

Hydrogen bonds are short-range interactions between hydrogen atoms of various groups, e.g., hydroxide (-OH), amine (-NH_2_), amide (-CONH-), and electronegative atoms having a lone electron pair (e.g., N, O, F). Although H-bonds are much weaker than covalent or ionic bonds, their collective nature promotes the self-assembling of the polymeric structures. They are critical for many biological and chemical systems and are also an important driving force to construct supramolecular hydrogels [1,8,9]. The strength of the hydrogen bonds is affected by the nature of constituent atoms, the bond geometry, and the environment. Therefore, the appropriate selection of gelators, consisting of a hydrogel, with various numbers and types of H-bond-forming groups, influences hydrogel mechanical properties and its interactions with biomolecules. The linearity of interactions, observed, for example, in DNA double-strand and protein β-sheet structures (Figure 1), gives stronger hydrogen bonds and also improves the gel strength [1,14]. Although H-bonds are stable in the presence of ions, pH changes may affect the intensity and strength of the bonds. The formation of competitive hydrogen bonds between gelator and water molecules may also cause deterioration of mechanical properties and even dissolving of the gel [8,9,11,14,15]. On the other hand, the ease of breaking and reformation of the hydrogen bonds gives the hydrogels self-healing and shear-thinning properties [1,16].

Various compounds are used to create hydrogels stabilized by hydrogen bonds. Many biopolymers belonging to the groups of proteins, polysaccharides, and nucleic acids, as well as synthetic polymers and small-molecular compounds, are used in this field. For example, the DNA strands can create strong hydrogels due to the formation of the multiple hydrogen bonds between base pairs. The molecules may be used as the backbone or cross-linkers for other molecules [1,8,10,17,18]. Other excellent, natural molecules for the preparation of H-bonds-based hydrogels are peptides and proteins. The hydrogen bonds are mainly formed between hydrogen and oxygen of the backbone amide groups. Both the long peptide chains and shorter, but more flexible, oligopeptides exhibit the ability to self-assemble in proper conditions. However, the mechanical properties of such gels are usually not satisfactory; thus, the proteins should be additionally chemically cross-linked or mixed with synthetic polymers. The ionization of amino acids depends on acidity/alkalinity of the environment, which is why the proteins are the most commonly used for pH-sensitive hydrogels’ preparation [10,11,19,20,21,22,23]. Another group of biopolymers that may create hydrogels stabilized by hydrogen bonds is polysaccharides (e.g., hyaluronic acid (HA), chitosan (CS), starch, agar) and their derivatives (e.g., carboxymethylcellulose, hydroxypropyl chitosan). The polysaccharides’ gel preparation often requires high temperature or a specially selected set of solvents (e.g., polar solvents, ionic liquids, and alkali/urea solutions) [24,25,26]. Hydrophilic synthetic polymers have also been widely investigated. Poly(vinyl alcohol) (PVA) can create self-healing, pH-sensitive hydrogels due to the formation of H-bonds and, additionally, through hydrophobic interactions. The PVA gels may be obtained by, for example, glycerol addition or a freeze-thawing process [16,27,28,29]. Hydrogels made of poly(vinyl pyrrolidone) (PVP) [12,30,31], poly(urethane), and poly(acrylamide) (PAAm) have also been extensively studied [1,9,32,33]. Small molecular multiple hydrogen bonding units, such as ureidopyrimidinone (UPy), benzene-1,3,5-tricarboxamide, and ureas, have also been investigated. They may be donors and acceptors of hydrogen bonds and can mediate in the creation of H-bonds between macromolecules [1,8,29].

### 2.2. Hydrophobic Interactions

Hydrophobic interactions are the second type of interaction, besides hydrogen bonds, which are crucial in the formation of biological structures, e.g., the tertiary structure of proteins. They can also be employed for the preparation of supramolecular hydrogels. Hydrophobic interactions are formed between the nonpolar moieties to minimize their contact with water. The interactions are relatively stronger than hydrogen bonds and Van der Waals interactions. The molecules, which form gels through hydrophobic interactions, usually possess hydrophobic and hydrophilic parts. The amphiphilic chains fold in water in such a way that the hydrophobic domains are in the core and surrounded by polar groups exposed to an aqueous environment. When the minimum gelling concentration is reached, the molecules aggregate, combine into micelles, and the gel is formed. The gel strength depends on the number, size, and geometry of hydrophobic domains. The properties can also be modified by surfactant or salt addition [34,35]. The ease of recreating hydrophobic interactions makes these gels exhibit excellent self-healing properties. The damaged hydrogel usually can be repaired at room temperature, regaining its mechanical properties [1,11,26,35,36,37,38]. The hydrogels which are formed by hydrophobic interactions often exhibit interesting negatively thermo-responsive behavior (the temperature increase causes the gelation process). Generally speaking, below the phase-transition temperature (Lower Critical Solution Temperature, LCST), the hydrophilic groups of the macromolecules are solvated by water molecules and bond with them by hydrogen bonds. Heating up the solution, the water molecules’ mobility increases and destroys the solvation sphere. Hydrophobic interactions become dominant. The macromolecules change conformation, which leads to aggregation, and as a result, the gel is formed [11,35,39,40].

**Figure 2:**
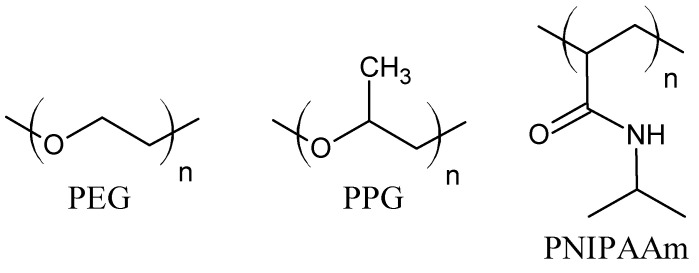
Molecular structure of poly(ethylene glycol) (PEG), poly(propylene glycol) (PPG), and poly(*N*-isopropylacrylamide) (PNIPAAm).

The hydrogels driven by hydrophobic interactions are composed of amphiphilic molecules. The balance between hydrophobic and hydrophilic moieties is crucial for hydrogel properties. For this purpose, hydrophobic units (e.g., aliphatic chains, aromatic rings, fatty acids [11,37]) are incorporated into hydrophilic polymer chains [38]. The hydrophobic moieties may be grafted onto the hydrophilic polymer chains, or micellar polymerization of monomers may be employed. During the polymerization, the hydrophobic unit is solubilized with a surfactant in aqueous solution and then copolymerized with a hydrophilic monomer, mostly by a free-radical mechanism [34]. Probably the most commonly investigated monomer for the fabrication of hydrogels based on hydrophobic interaction is *N*-isopropylacrylamide. The poly(*N*-isopropylacrylamide) (PNIPAAm) contains hydrophilic amide groups and hydrophobic isopropyl chains (Figure 2). Its low critical solution temperature (LCST) is about 32 °C, close to body temperature. The PNIPAAm thermoresponsive properties can be easily modified through copolymerization with more hydrophobic or hydrophilic units, e.g., *N*-alkyl-, polycaprolactone (PCL), poly (acrylic acid) (PAA), poly(ethylene glycol) (PEG), proteins, and polysaccharides [1,7,11,39,40,41]. Triblock copolymers, poly(ethylene oxide)-poly(propylene oxide)-poly(ethylene oxide) (PEO-PPO-PEO) (Figure 2), known also as Pluronics or a Poloxamers, form micelles with the temperature increase. The hydrophobic PPO chains are wrapped inside an inner core, while the hydrophilic PEO segments are exposed outside the micelles to an aqueous medium. Moreover, increasing the copolymer concentration above the critical micellar concentration causes further interactions between micelles and gel stiffening [39]. Furthermore, the copolymerization of PEG with polyesters, e.g., poly(lactic acid) (PLA) and poly(lactic acid-co-glycolic acid) (PLGA), or hydrophobic protein domains can lead to the formation of thermosensitive hydrogels due to hydrophobic interactions [1,35,36,39]. The multi-block copolymer of PEG and dimer of fatty acid (DFA) was also investigated. In water, the hydrophobic DFA self-aggregate to micellar domains, which work as cross-linking bonds. The PEG–DFA hydrogel was also used as a matrix for carbon nanotubes, and the result was a shear-thinning nanocomposite hydrogel with increased electrical conductivity [37,42]. The polysaccharide hydrogels, cross-linked via hydrophobic interactions, were formed on the base of carboxymethylcellulose (CMC) and hydrophobic moieties, such as dioctylamine or dodecylamine. The grafting of methylcellulose with *N*-isopropylacrylamide in various ratios gives hydrogels with tunable thermosensitive properties. It has also been observed that chitosan in combination with β-glycerophosphate gains negatively thermo-responsive properties [34,39].

### 2.3. Ionic Interactions

Ionic interactions are based on the electrostatic attraction of oppositely charged ions/groups. An ion can also interact with a dipole (polar molecule/group) or induce polarization of a non-polar molecule (induced dipole). The interaction between cationic (e.g., protonated amines) and anionic (e.g., carboxylates, sulfates) functionalities of polymers, as well as charged functionalities of polymers and oppositely charged ions, are used to obtain hydrogels. The ionic hydrogel formation and its properties depend on polymer concentration, ionization degree of the polymer cationic and anionic groups, pH, ionic strength, temperature, and time of interaction. The gels are easily obtained, often by a one-step mixing procedure, although controlling this process is often challenging. Because of the presence of charged groups, the ionically bonded hydrogels are highly sensitive to pH changes and salt concentration in swelling solution, which makes them attractive for drug and active-substance delivery [1,26,43,44].

**Figure 3:**
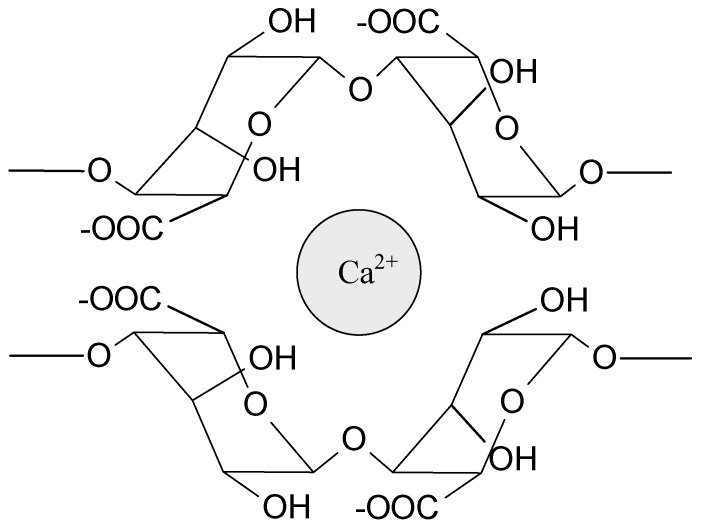
Schematic representation of the ionic interaction of calcium ion with alginate.

Sodium alginate (SA) is probably the most widely tested biopolymer cross-linked by interactions with ions. These polyanionic molecules can form hydrogels by an interaction with divalent (e.g., Ca^2+^, Sr^2+^, Ba^2+^) and trivalent metal cations (e.g., Fe^3+^, Al^3+^). Calcium ions are the most often used to prepare alginate-based hydrogels for medical applications (Figure 3). The source of Ca^2+^ (e.g., CaCl_2_, CaSO_4_, CaCO_3_) influences the gel strength and homogeneity. Interestingly, magnesium ions do not induce electrostatic interaction with alginates. Due to the high strength of the interactions, these hydrogels are relatively durable and stable, but they do not exhibit self-healing properties. Alginate can also electrostatically interact with polyelectrolytes such as chitosan [1,36,43,45]. Chitosan, as a positively charged polymer, willingly interacts with anionic molecules (phosphate salts, carboxylate salts) and creates polyelectrolyte complexes with polysaccharides (e.g., pectin, dextran, carboxymethyl cellulose, hyaluronic acid) and proteins (e.g., gelatin), as well as synthetic polymers, e.g., poly(acrylic acid) [36,43,46]. The ionically bonded hydrogels prepared from ampholytic polymers, e.g., proteins, poly(acrylamide), and poly(methacrylate) derivatives, have also been investigated [43,47,48,49]. The ionic interactions are present in many different systems and very often stabilize hydrogel structure, cooperating with other types of interactions.

### 2.4. Metal–Ligand Coordination

Hydrogels based on metal–ligand coordination are widely studied because of their interesting tunable properties. In a coordination bond, both electrons originate from the same atom. Moreover, more than one donor group (ligand) may be involved in bond formation with the central metal ion. The strength of the coordination bond varies over a wide range and can be comparable or even higher than the strength of the covalent bond, but at the same time, the bond remains more dynamic and reversible. This makes the metal–ligand-based materials exhibit toughness, good adhesion, as well as shear-thinning and self-healing ability. Due to the various organic ligands conjugated to polymer backbones or copolymerized with other monomers, the linear, branched, dendritic, or star-shaped complexes can be formed [26,36,50,51,52].

**Figure 4:**
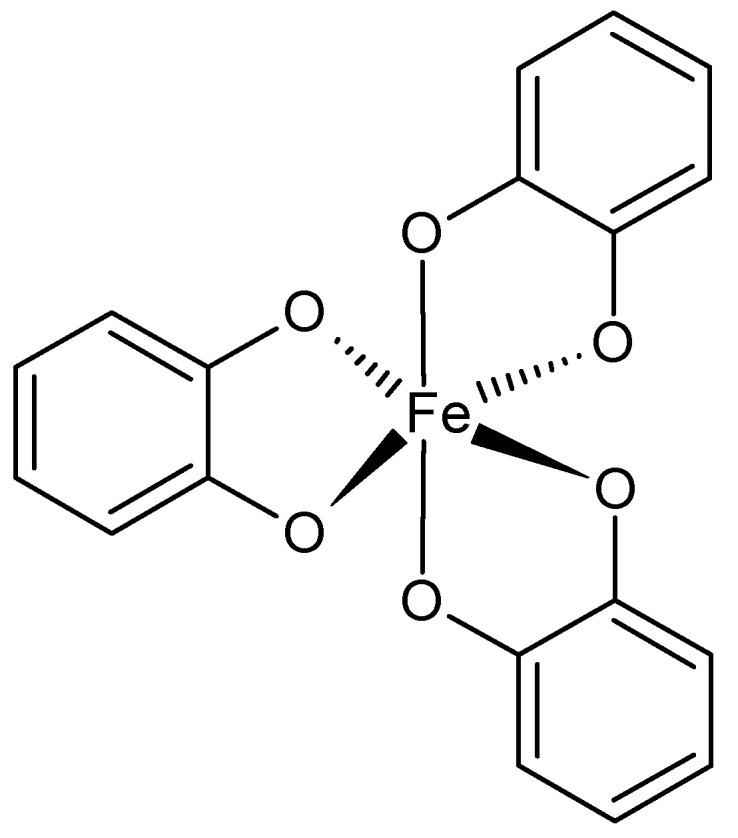
Catechol-Fe^3+^ coordination.

The ferric ions and catechol ligands are willingly employed for supramolecular, adhesive hydrogels preparation (Figure 4). This complex was found in mussels’ adhesive proteins that provide good adherence to different wet surfaces. The catechol also interacts with boron ions, forming pH-sensitive hydrogels. The catechol ligand has been incorporated into, for example, PEG, PAA, PPO-PEO copolymers, PNIPAAm, modified SA, and chitosan. The catechol-containing hydrogels possess self-healing ability and are highly elastic [48,49,50,53,54]. Histidine is another useful ligand present in peptide sequences or conjugated with polymers, e.g., PEG. Usually histidine forms complexes with zinc ions. Depending on the amount of histidine, for example, in the sequence of the polypeptide, toughness of the hydrogel can be improved while maintaining flexibility and self-healing [50,51,52,55]. Bisphosphonates (BPs) are a family of phosphoroorganic molecules that exhibit a high affinity for calcium ions. For this reason, they are useful in the preparation of bone-targeting drug carriers. They can also chelate different metal ions, such as Cu^2+^, Zn^2+^, and Mg^2+^. BPs can be grafted to biocompatible polymers such as PEG and hyaluronic acid [50,56]. EDTA, ethylenediaminetetraacetic acid, is a well-known ligand interacting with divalent ions (Ca^2+^, Mg^2+^, Fe^2+^). Its ability to coordinate metal ions is due to the presence of four carboxyl and two amine groups. It has been conjugated to polymers such as PVA. It was also an inspiration for developing hydrogels based on Fe^3+^ ions and poly(acrylic acid) or its copolymers [50,52,53,57].

### 2.5. Host–Guest Interactions

Another type of interaction used for the fabrication of supramolecular hydrogels is the host–guest interaction. The inclusion complex between the macrocyclic, containing a cavity, host molecule and the suitable guest molecule is formed. The clue of this interaction is the complementary size and shape of the host cavity and guest molecule. Both moieties may be engaged via non-covalent interactions, such as hydrogen bonds, Van der Waals, hydrophobic, electrostatic interactions, or coordination bonds. In this way, cavitands may be paired with various guests, including drugs, biomolecules, and polymers, which are both inert and stimuli-responsive. It is relatively easy to incorporate the host–guest moieties into the hydrogel structure, and the stoichiometry of this interaction is precisely defined (one host cavity can hold one guest molecule). This provides greater predictability and reproducibility of hydrogel properties. The reversibility of the host–guest interaction gives the hydrogels self-healing and shear-thinning properties [11,36,58,59,60].

**Figure 5:**
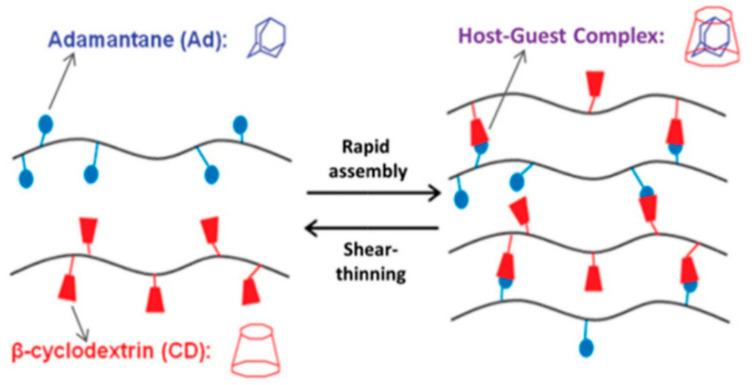
The host–guest interaction between β-cyclodextrin and adamantine. Reprint with permission [36]; Copyright 2019, Wiley.

The group of hosts cavitands includes a variety of naturally-derived and synthetic macrocycles and their derivatives. Cyclodextrins are the most important representative of the first group. These are water-soluble, low-toxicity cyclic oligomers, most commonly formed of 6, 7, or 8 α,D-glucopyranose units (α-, β- and γ-CD, respectively) in the shape of a truncated cone. They have a hydrophilic external and relatively hydrophobic inner cavity. The small-cavity, α-CD, incorporates mainly linear guest molecules, while β-CD interacts with adamantane, azobenzene, ferrocene, cholesterol, and PEG (Figure 5) [13,36,58,59,60,61,62,63,64]. The CD units can be incorporated into cyclodextrin-polymers (e.g., polyrotaxanes), as well as being grafted to other polymer chains, e.g., alginate, HA, PEG, and PAA. Then, the guest molecules are attached to another polymer chain, and this promotes gel formation [11,13,59,60,61,62,63]. Among the synthetic macrocyclic cavitands are cucurbiturils, crown ethers, calixarenes, and pillararenes [58]. The hydrogels based on cucurbit[n]urils (CB) are the most studied. CB contains 6, 7, or 8 glycoluril monomers linked by methylene bridges into a pumpkin-shaped structure. The hydrophobic cavity is bordered by carbonyl groups. For this reason, CB strongly interacts with various guest-molecules, e.g., amino acids, peptides, ammonium groups, or aliphatic amines. The CB can be easily bonded to polysaccharides, e.g., alginate, HA, carboxymethyl cellulose, and hydroxyethyl cellulose. The formation of micelle-like aggregates of sodium alginate in a water solution after CB addition has also been observed [36,58,65].

The comparison of strength and range of the non-covalent interactions is reported in a summarized way in Table 1.

## 3. Supramolecular Hydrogels as Carriers for Biologically Active Substances

Hydrogels have been extensively investigated for their application as carriers for active substance delivery systems [5,66,67,68]. These materials have attracted considerable attention, particularly in solutions proposed for the controlled release of drugs, as bioadhesive implements, or as carriers of therapeutic agents to the target sites. Hydrogel-based materials with incorporated active substances can be intended for oral, epidermal, and subcutaneous application, as well as being used to deliver drugs by rectal and ocular routes. The most investigated entrapment of drugs and other therapeutics are polymeric hydrogels based on covalent linkages. Numerous such hydrogels containing drug complexes have successfully been developed for skin diseases [69,70], wound healing [71,72], and inflammatory alleviation [73], among many others.

**Figure 6:**
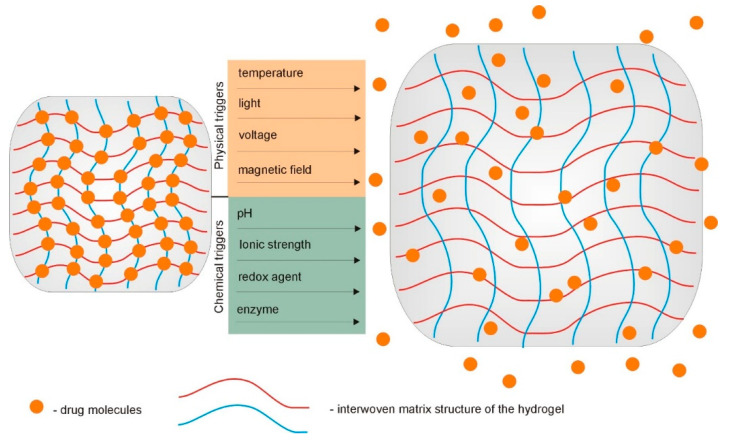
Hydrogel-based implantable therapeutic carriers for local therapy operate as swelling-controlled systems in response to various chemical and physical triggers.

Over the past years, rapid progress in the field of supramolecular hydrogels, with a vast array of tunable properties as drug carriers, has been observed [74,75,76]. As described above, due to the interesting physicochemical characteristics and peculiar functions, supramolecular hydrogels are being widely explored as carriers for different biologically active substances. Their ability to undergo reversible swelling, gel–sol transition under the influence of changes in the relevant environmental stimuli, and their injectability are highly valued. Site-specific controlled release of drugs is one of the most important issues in current therapeutics. The use of stimuli-responsive hydrogels as drug delivery systems enables the programmed delivery of a pharmaceutically active substance. In response to specific stimuli, such as temperature and pH, these hydrogels can control the delivery of loaded therapeutic agents into a specific place in the body (Figure 6). The increasing popularity of supramolecular gels and their advantage over fully covalently crosslinked hydrogels results primarily from the possibility to manage the appropriate reversible non-covalent interactions in the molecular structure of the gelators [1,11,77].

The supramolecular hydrogels have found applications in drug delivery, antimicrobial therapy, gene transfection, and tissue engineering. Physical gels are important, especially in controlled-release applications, and their use in this field has experienced a strong growth in recent years.

### 3.1. Supramolecular Hydrogels as Drug Delivery Systems

#### 3.1.1. Supramolecular Hydrogels for Cancer Drug Delivery

**Figure 7:**
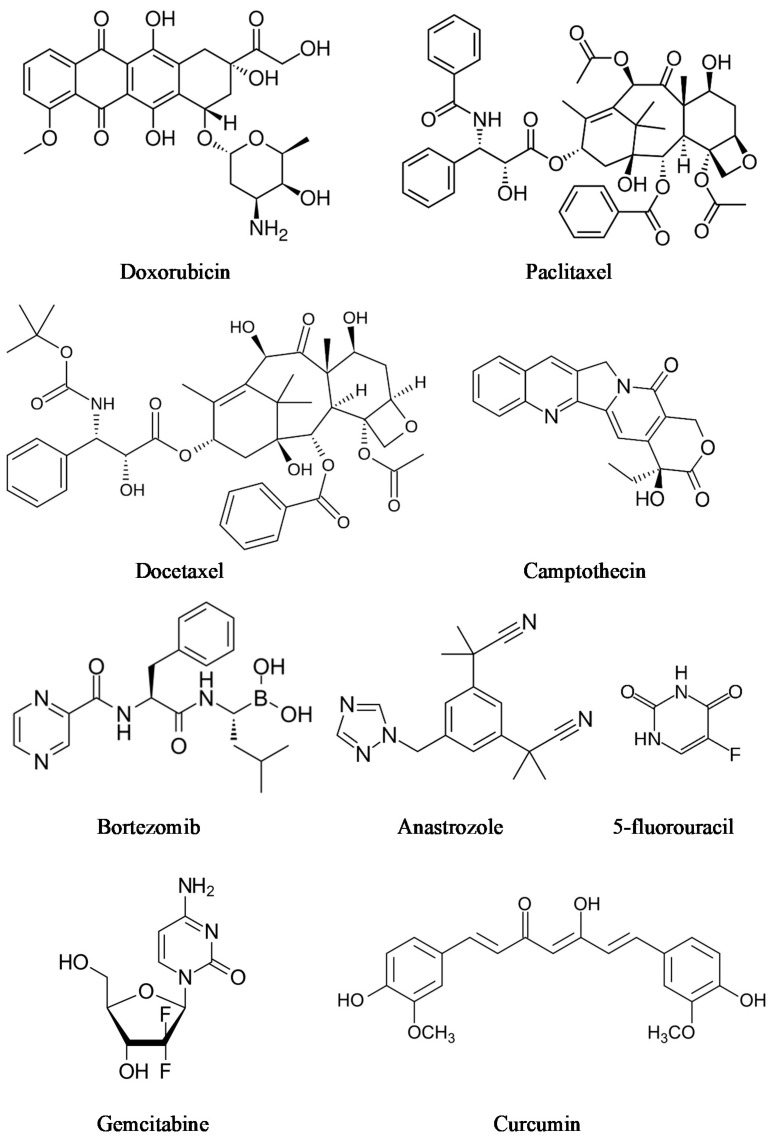
Anti-cancer agents.

Supramolecular hydrogels are very demanded in medicine. Various types of supramolecular polymeric systems have been proven to treat different types of cancer [78,79,80]. Cancer is one of the world’s most extensive health problems, because cancer incidence and mortality are growing every day [81,82]. Traditional intravenous chemotherapy, one of the most common types of cancer treatment, can have adverse effects, such as myelosuppression, liver or kidney dysfunction, and neurotoxicity [83]. To conquer the limitations associated with conventional chemotherapy, injectable hydrogels and local chemotherapy that efficiently avoids side effects, due to releasing drugs locally at the tumor site, can be promising alternatives for cancer treatment [84]. There are many cancer drugs and cancer drug combinations (Figure 7). Chemotherapeutics consist of a large group of drugs, including doxorubicin, docetaxel, camptothecin, 5-fluorouracil, paclitaxel, gemcitabine [85], bortezomib [86], and anastrozole [87]. Moreover, several natural compounds, such as magnolol [88,89] and curcumin [90,91], have proven their potential against cancer in preclinical studies.

A review of supramolecular hydrogels, including their uses as carriers of anti-cancer agents for in vitro and in vivo cancer therapy, is presented in Table 2.

Various drug delivery systems based on environmentally sensitive nanocarriers that can be injected instead of surgically implanted were developed as a promising choice for local chemotherapy and cancer management [107,108]. Smart hydrogels exhibit a very effective drug release with long-term local retention. These carriers can also possess high drug loading. Moreover, drug toxicity is localized in the tumor site [109].

Peptide-based supramolecular hydrogels have been satisfactorily designed as promising anticancer drug carriers. Self-assembling biocompatible peptide hydrogels are characterized by very effective drug loading, ensuring their high content as well as sustained release profile. Truong et al. prepared a hydrogel made of phenylalanine–phenylalanine dipeptide [FF, (F: phenylalanine)] bearing the Fmoc (9-fluorenylmethyloxycarbonyl) group, which is one of the most typical protecting groups. Hydrogel (Fmoc-FF) had entrapped anti-tumor medicaments paclitaxel and 5-fluorouracil [110]. The cytotoxicity of obtained samples was tested on three different cell lines: HeLa, Caco2, and HGF-1 cells. This study proved that in vitro biological activity of self-assembled systems, such as Fmoc-FF gels, in which the gel stability is the main criterium influencing their potential applications as drug carriers, needs to be estimated with wariness to avoid the misinterpretation as false-positive results. A tripeptide-based thixotropic hydrogel, Boc-FFF-COOH (BOC: tert-butyloxycarbonyl protecting group), with a permuting L and D configuration for releasing anticancer doxorubicin at a physiological pH and temperature, was designed and prepared by Basu et al. [111]. Doxorubicin was successfully encapsulated in hydrogels made of compounds LLL, DLL, DDD, and LDD. Generally, the incorporation and location of D-residues determines the properties of obtained supramolecular hydrogels, including their stiffness and drug-release capacity, which enables the optimization for designing future drug delivery carriers. Numerous peptide-based supramolecular hydrogels have been successfully designed by using a peptide with higher length. Among others, the supramolecular naphthalene-GFFYEE-catechol hydrogel contains bortezomib [112], an H_2_O_2_-responsive hexapeptide-based hydrogelator bearing the thiazolidinone group with gemcitabine [113], and supramolecular peptide amphiphile nanofiber gels (Lauryl-VVAGEEE-OH and Lauryl-VVAGKKK-Am) with doxorubicin, which were encapsulated within the gels [114].

A novel nanomaterial based on short peptide and curcumin as a therapeutic agent, with promising and potential therapeutic options for liver tumor-targeting therapy, was designed and synthesized by Chen et al. [115]. This research group developed a glycyrrhetinic acid-modified curcumin supramolecular hydrogel. The obtained nanocarrier was an ideal candidate for hepatocellular carcinoma therapy due to enhanced cellular uptake and more efficient inhibition capacity to HepG2 cells compared to a control compound.

#### 3.1.2. Supramolecular Anti-Inflammatory Hydrogels

Supramolecular gels have been studied not only for applications in cancer therapy. Limón et al. prepared supramolecular gels from a bis-imidazolium-based amphiphilic molecule in ethanol–water mixtures, in which anionic anti-inflammatory drugs, ibuprofen sodium salt, and indomethacin, were entrapped [116]. Significant differences could be seen in the hydrogel structure depending on the incorporated drug; hydrogel alone is formed, comprising entangled fibers of ~100 nm in width, while after the incorporation of ibuprofen, thick and stick fibers with diameters in the range of 300–1500 nm were observed. Hydrogels containing indomethacin had small groups of much shorter fibers that were stick longitudinally. However, the excellent stability of the hydrogels was comparable, regardless of the used drug. Drug release and skin penetration profiles suggest that obtained materials, especially indomethacin-incorporated gel, can offer applications for delivering poorly water-soluble drugs for skin diseases therapies, including acute and chronic inflammation.

Curcumin has received worldwide attention, mainly due to its antioxidant and anti-inflammatory properties [117]. Kumar Vemula et al. developed a model system for the controlled delivery of anti-inflammatory curcumin by an enzyme-triggered drug release mechanism during the degradation of encapsulated hydrogels [118]. The curcumin was encapsulated in the self-assembled hydrogel, in which the gel fibers were stabilized by intra- and intermolecular hydrogen bonding, π-π stacking, and Van der Waals interactions. By manipulating the concentration of enzyme or temperature, the enzyme-triggered hydrogel degradation was performed to control the release of the entrapped drug into the solution at bodily temperature. Its novel delivery model for hydrophobic drugs could be utilized in pharmaceutical research for developing controlled drug-delivery systems from sustainable resources. Zhou et al. prepared a curcumin-loaded supramolecular hydrogel, composed of α-CD and metoxy poly(ethylene glycol)-block-poly(ε-caprolactone) (MPEG-PCL) as carrier material for inflammatory skin treatment [119]. In vivo results confirmed that the curcumin-loaded supramolecular hydrogel displayed better anti-inflammatory effects than dexamethasone ointments against croton oil-induced ear edema.

### 3.2. Supramolecular Hydrogels for Antimicrobial Properties

The World Health Organization has rated the antimicrobial resistance crisis as a priority health issue, because the treatment of many diseases caused by microorganisms, such as bacteria, fungi, and viruses, after developing effective drugs has become problematic again. The rapid emergence of drug-resistant microbes has taken place worldwide due to the misuse of antibiotics in humans and animals, including the overuse of these drugs [120]. Nowadays, the consequences of antimicrobial resistance are alarming [121]. Moreover, due to the dynamic situation, creating new antibiotics is not profitable for the pharmaceutical industry. Unfortunately, after many years of success in controlling many diseases, bacterial infections, such as pneumonia, tuberculosis, and gonorrhea, have become a threat. The traditional medicines that effectively fought against the development of pathogens responsible for these diseases have become ineffective [122,123]. Current challenges in treating human immunodeficiency virus (HIV) are caused by the rising use of antiretroviral treatment and, consequently, high variability of HIV. Drug-resistant mutations contribute to antiretroviral treatment failure [124]. Developing antimicrobial agents with novel mechanisms of action has become an urgent need to solve the global problem of antibiotic resistance. The design of supramolecular systems for antimicrobial therapy is receiving increasing attention from scientists.

#### 3.2.1. Supramolecular Hydrogels with Antibacterial Activity

Tuberculosis affecting the lungs is another difficult-to-treat disease that scientists working on synthesizing new drug carriers using supramolecular chemistry want to address. More et al. proposed a graphene-based hydrogel with entrapped para-aminosalicylic acid and pH-sensitive properties, which could be potentially used to manage *Mycobacterium tuberculosis* [125]. The sonification method was used to prepare a supramolecular self-assembly hydrogel. Hydrogen-bonding interactions between surface groups of graphene oxide and functional groups of para-aminosalicylic acid occur during self-assembly gel formation. The obtained hydrogel was biocompatible and, most importantly, strongly indicated in vitro cytotoxicity against MCF-7 cells, as well as antimicrobial properties against *Staphylococcus aureus* and *Escherichia coli*.

**Figure 8:**
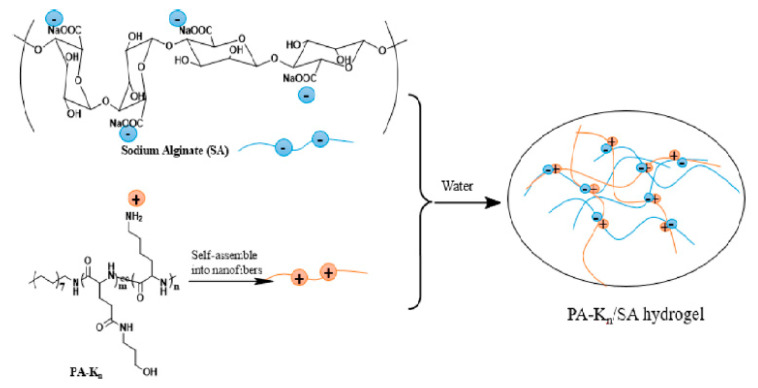
Cationic peptide amphiphiles-based supramolecular hydrogels with sodium alginate (n—the quantity of lysine unit in the peptide segment). Reprint with permission [127]; Copyright 2017, American Chemical Society.

Many short peptides gelators that demonstrate antibacterial properties have the ability to self-assemble into the form of supramolecular hydrogels [1,122,126]. Moreover, peptide-based antibacterial hydrogels are characterized by good biocompatibility. It is also possible to modify their structure and other properties, especially antibacterial efficiency. Wan et al. obtained a series of cationic peptide amphiphiles (PA) that could self-assemble into hydrogels [127]. The obtained hydrogels contained lysine, which exhibits pH-responsive action and antibacterial activity, as well as sodium alginate (SA) as a gel strengthening agent (Figure 8).

Several reports on the synthesis and properties of the Fmoc-based supramolecular hydrogels with antibacterial properties have recently appeared. The first scientific report about the formation of hydrogels with Fmoc-protected amino acids and dipeptides was published in 1995 [128]. Since then, supramolecular hydrogels, prepared from peptides or amino acids attached to adjacent Fmoc, are of interest to many research groups [129]. A hybrid supramolecular hydrogel based on Fmoc-FF and fullerene (C60 pyrrolidine tris-acid, C60-PTC) was designed by Zhang et al. [130]. Both components of the self-assembled hydrogel exhibited synergistic effects due to many different non-covalent interactions between them. An improvement in the mechanical properties of the hydrogel was observed, which made this formulation suitable for injectable applications. The Fmoc-FF/C60-PTC hydrogel proved to be effective during photodynamic antibacterial tests performed on *Staphylococcus aureus*.

Xie et al. [131] reported amino acid-based hydrogels that were obtained by self-assembly of Fmoc-tryptophan (Fmoc-W), Fmoc-methionine (Fmoc-M), and Fmoc-tyrosine (Fmoc-Y). The obtained materials were tested for antibacterial activity. The results showed that all hydrogels demonstrated antibacterial activity against Gram-positive bacteria, and Fmoc-W hydrogel had the best efficiency (95.3% inhibition of *S. aureus*). In comparison, Fmoc-Y hydrogel was the least effective (57.3%). However, their antibacterial activity against Gram-negative bacteria was slight. In addition, significant differences in the nanostructure of the prepared hydrogels were observed, which impacted the results obtained in the anti-bacterial activity tests. In the case of Fmoc-M and Fmoc-Y, their nanofibers were flexible. However, Fmoc-W had stiffer and more aligned nanofibers in the 3D network. As a potential carrier for drug delivery applications, self-assembling Fmoc modified phenylalanine (Fmoc-F) hydrogels have also been synthesized, and their antibacterial properties have been characterized [129,132,133].

An interesting idea is incorporating inorganic particles in the form of nanohydroxyapatite (nHAp) into the hydrogel structure. Li et al. prepared Fmoc-F-based supramolecular hydrogels containing nHAp [134]. Apart from the improvement of the mechanical parameters of the hydrogel, no negative influence of the addition of inorganic nanoparticles to the antibacterial properties of the hydrogel was observed. Furthermore, the release profile of chlorogenic acid proved a satisfactory inhibition result of *S. aureus*.

#### 3.2.2. Supramolecular Hydrogels for HIV Antiretroviral Therapy

Li et al. reported novel multifunctional enzyme-responsive supramolecular hydrogels for sustained release of anti-HIV drugs, representing a new approach and making an important contribution in antiviral therapeutics [135]. This research group proposed self-assembled, anti-inflammatory, and anti-HIV hydrogels containing anti-inflammatory naproxen, as well as lamivudine (3TC) and zidovudine (AZT), as analog-reverse transcriptase inhibitors against HIV. The phosphate group was also included in the hydrogelator structure, which ensured hydrogelation at a definite physiological pH. Moreover, the phosphate group’s presence permitted the increase of the hydrogel’s viscoelasticity upon the treatment of phosphatase. The solution suggested by Li et al. is the answer to challenges in HIV prevention, because gels based on conventional polymers have not shown good effectiveness in HIV therapy so far. HIV, the virus that causes AIDS (acquired immunodeficiency syndrome), is still a major global health emergency.

### 3.3. Supramolecular Hydrogels for Controlled Gene Delivery

Numerous types of supramolecular systems, especially cationic supramolecular hydrogels, have been successfully designed and developed for gene delivery as potential nonviral vectors for in vitro or in vivo gene expression [8]. These systems can condense and transfer genetic material to a concrete location to gain a therapeutic effect. Controlled gene transfer vectors target the tumor cells or tissue and play an important role in future cancer therapy. This strategy has the advantage over conventional treatment for cancer, including a high therapeutic dose without risking systemic adverse effects and being cost-effective in the long run, because most gene therapies are single-time applications [136,137].

Supramolecular hydrogels based on cyclodextrins have garnered a lot of attention as systems for gene-delivery approaches (Table 3). CD-based polypseudorotaxane hydrogels are characterized by interesting properties, including thixotropic, biocompatibility, and easy modification. Therefore, they are suitable for use as injectable drug or gene delivery systems, whereas CD-based host–guest supramolecular hydrogels can potentially be applied for stimuli-responsive drug/gene carriers [138,139].

Motoyama et al. demonstrated polypseudorotaxane systems (PPRXs) that were based on a PEGylated α-cyclodextrin/polyamidoamine dendrimer conjugate with α- or γ-CD as novel sustained-release carriers for pDNA in vitro and in vivo (PEG-α-CD/pDNA; PEG-α-CDE/pDNA/α-CyD PPRX; PEG-α CDE/pDNA/γ-CyD PPRX) [140]. As the authors noted, the molecular hydrogels obtained by them had many advantages, such as excellent efficacy of encapsulation of pDNA or other nucleic acids, as well as the possibility to use them for other pegylated carriers, such as microspheres and microcapsules.

A cationic block copolymer based on Pluronic F-68 and poly(L-lysine), which interacts simultaneously with α-CD by the host–guest inclusion action, was synthesized and characterized by Ma et al. [141]. The content of hydrogel components had a significant impact on gelation time, mechanical strength, and release of the encapsulated plasmid DNA. Therefore, the properties of the obtained supramolecular hydrogel could be relatively easily modified. What is most important is that the plasmid DNA complexes released from the hydrogel had a sustained gene transfection ability. An in vitro cytotoxicity assay on mouse fibroblast cells, 3T3, confirmed their biocompatibility.

**Figure 9:**
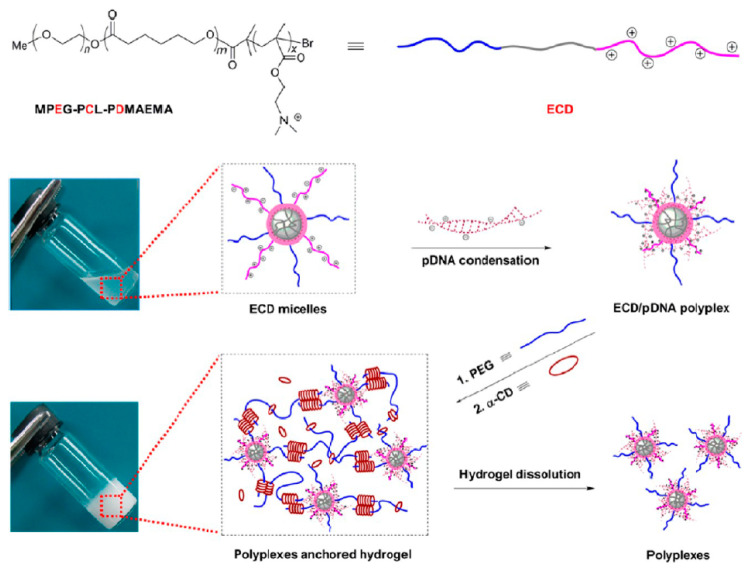
Schematic illustration for obtaining ECD/pDNA anchored α-CD/PEG supramolecular hydrogels (ECD corresponds to the MPEG-PCL-PDMAEMA, where E: MPEG, C: PCL, and D: PDMAEMA). Reprint with permission [142]; Copyright 2012, American Chemical Society.

Cyclodextrin-based injectable supramolecular hydrogel systems, as sustained gene delivery carriers, were formed by Li et al. [142]. MPEG-PCL-PDMAEMA copolymers were prepared to condense pDNA, and the obtained hydrogels were suitable to release the pDNA in the form of stable polyplexes and in a sustained manner for up to six days (Figure 9).

Lin et al. prepared injectable hydrogels by α-CD and PEGylated arginine-functionalized poly(L-lysine) dendron (MPEG-PLLD-Arg) through the host–guest interaction [143]. In vivo results suggested that the pMMP-9-loaded hydrogel was effective in tumor gene therapy by providing a long-term, sustained tumor site treatment.

Likewise, Liu et al. reported interesting results in the field of supramolecular hydrogels for sustained, in vivo gene delivery of anionic plasmid DNA at therapeutic levels, with the simultaneous release of chemotherapeutic paclitaxel, which might be beneficial for further applications in personalized medicine [144]. The authors designed and synthesized the carrier for drug and gene sustained release in the form of the injectable supramolecular hydrogel by complexations between α-cyclodextrin and the cationic MPEG-PCL-PEI-FA copolymer. The obtained complex containing paclitaxel and plasmid DNA could self-assemble into nanoparticles with size ~230 nm. The formed supramolecular hydrogel had the ability to co-deliver the chemotherapeutic drug and Nur77 gene to combat Bcl-2-overexpressed therapeutic-resistant tumors in a targeted manner.

Another drug and gene co-delivery system for high-efficiency cancer treatment was developed by Ma et al. [145]. The authors proposed an approach by localized, sustained co-delivery of PLK1shRNA/polylysine-modified polyethylenimine complexes (PLK1shRNA/PEI-Lys) and doxorubicin for the treatment of osteosarcoma in vitro and in vivo. In this method, doxorubicin and PLK1shRNA/PEI-Lys were loaded into a biodegradable PLGA-PEG-PLGA hydrogel. The results proved that PLK1shRNA/PEI-Lys in the hydrogel lead to a significant increase of the anti-tumor effect of doxorubicin, which making this injectable material a potential candidate for efficient clinical treatment of osteosarcoma. The synergistic antitumor effects included tumor growth suppression, silencing of the PLK1 gene, promoting tumor apoptosis, as well as the effect on cell cycle regulation.

### 3.4. Supramolecular Hydrogels in Tissue Engineering

Tissue engineering is a branch of regenerative medicine that uses cells and other materials to either enhance or replace damaged biological tissues. The regeneration of tissue defects is potentially possible by culturing a patient’s cells on a polymer matrix, which is the scaffold for new tissue [146,147]. Due to the structural similarity to the macromolecular components in the extracellular matrix, supramolecular hydrogels are ideal candidates as media for tissue engineering. Moreover, these materials can provide a suitable biological environment for encapsulating bioactive molecules, such as growth factors and cells [148].

Stimuli-responsive supramolecular hydrogels have been fully examined for applications in this field of medicine because of their advantage of changing their physicochemical properties in response to suitable stimuli, such as temperature or light, allowing modulation of the cell microenvironment [11,15]. Numerous studies have looked at temperature-responsive supramolecular hydrogels. For example, Hong et al. prepared a poly(ethylene glycol)-b-poly(L-alanine) thermo-gelling system as an injectable 3D culture system, by incorporating tonsil-derived mesenchymal stem cells (TMSCs), tauroursodeoxycholic acid (TUDCA), hepatocyte growth factor (HGF), and fibroblast growth factor 4 (FGF4) [149]. By increasing the temperature to 37 °C, the system underwent thermal gelation. The obtained polypeptide thermogel was characterized by pronounced expressions of the hepatic biomarkers, which makes this material promising for tissue engineering applications. An example of a light-responsive physical hydrogel is that synthesized by Rosales et al. [150]. This research group prepared a hyaluronic acid-based hydrogel via host–guest complexation between azobenzene- and α-CD-containing HA chains, with NIH 3T3 fibroblasts encapsulated into the hydrogels. This photo-responsive system had variable crosslink density and mechanical stiffness, depending on the defined wavelengths of light. The controlled light exposure lead to a dynamic hydrogel, suitable for tissue engineering.

**Figure 10:**
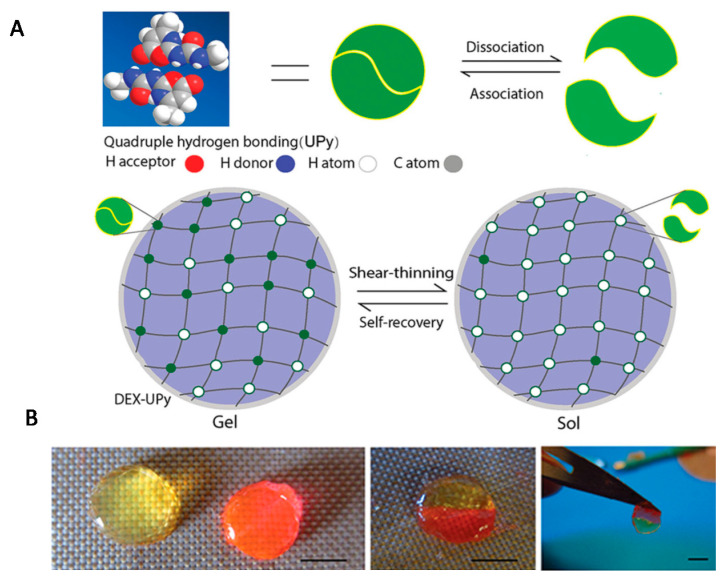
(**A**) Scheme of the experimental strategy for obtaining a DEX-UPy hydrogel with shear-thinning and self-integration properties; (**B**) visualization of these properties (scale bar corresponds to 5 mm). Reprint with permission [152]; Copyright 2015, Wiley.

Numerous scientific reports have focused on the use of molecular hydrogels in the regeneration of cartilage and bone [1,29,151]. Hou et al. prepared an injectable supramolecular hydrogel based on dextran by grafting a significant number of multiple-hydrogen-bond (ureidopyrimidinone) [152]. The UPy unit is a quadruple hydrogen-bonding array, which is a driving force for hydrogel formation due to strong hydrogen bonds between the dextran strands. By changing the ratio of UPy to dextran, the UPy graft density could be controlled, and hence, the formation of supramolecular hydrogels of varying stiffness was possible. The obtained hydrogel had self-integrating and shear-thinning properties (Figure 10). Chondrocytes for cartilage formation and rabbit bone marrow stem cells (BMSCs) were encapsulated separately into the hydrogeland subsequently, hydrogels were implanted subcutaneously in a nude mouse. In vivo results confirmed that both cartilage and bone tissues were successfully regenerated.

Feng et al. [153] designed and prepared novel injectable carriers of therapeutic cells and drugs to assist the repair and regeneration of bone, cartilage, and tendon, using gelatin crosslinked by weak host–guest interactions, through a novel host–guest macromonomer (HGM) method. The hydrogel was obtained due to complexation between aromatic residues of gelatin and the free-diffusing photocrosslinkable acrylated β-CD. The most important advantages of their proposed system were mechanical strength, controlled release of a small hydrophobic molecule, and supporting cell retention, as well as the injection capacity and re-moldable properties. In addition, it is worth noting that this host–guest supramolecular macromer significantly enhanced the osteogenic differentiation of the encapsulated human mesenchymal stem cells (hMSCs) in comparison with the conventional chemically crosslinked methacrylated gelatin (MeGel) hydrogel. In a further study, this group investigated the gelatin HGM hydrogels for the long-term chondrogenesis of human BMSCs after injection of the material into defects in rats’ knees [154]. Fully regenerated cartilage in the defect site was observed six weeks after the implantation, making this hydrogel a promising carrier of therapeutic cells and drugs for cartilage regeneration.

Supramolecular hydrogels cross-linked by metal cations have also been applied in cartilage tissue engineering, specifically as tissue adhesives. An interesting example is the research by Fan et al., who used genipin (long-term acting crosslinker) and Fe^3+^ (rapid crosslinker) to obtain a double crosslink tissue adhesive (DCTA), comprising of a dopamine-conjugated gelatin macromere [155]. It turned out that the obtained DCTA hydrogel was very effective: when compared to the commercial fibrin glue, it showed 24 times stronger action. Shear test results indicated that in the case of 2-hour-curing, cartilage gluing strength was significantly increased from 8.0 kPa to 194.4 ± 20.7 kPa for DCTA and the commercial fibrin glue, respectively. Moreover, DCTA could well support hMSCs adhesion and proliferation. In vivo biocompatibility and biodegradability were confirmed after implantation of DCTA in the subcutaneous tissues of the nude mice. It can be concluded that this novel DCTA material may be a highly promising product as an adhesive glue for cartilage tissue repair.

## 4. Conclusions

The presented hydrogels based on various non-covalent interactionssuch as hydrogen bonds, hydrophobic interactions, electrostatic interactions, metal–ligand coordination, and host-gest interactions have many interesting features, useful in drug and active substances delivery. Due to these reversible interactions, supramolecular hydrogels have many advantages including shear-thinning and self-healing properties, good mechanical properties, and sensitivity to stimuli. These unique properties have driven notable advances in the field of controlled drug delivery systems. Scientific research on the design and use of hydrogels as carriers for anti-cancer, anti-microbial, and anti-inflammatory drugs is very advanced. Recent successes in the delivery of therapeutic nucleic acids into target cells, especially in the treatment of tumors, have demonstrated the potential of supramolecular hydrogels as carriers in gene therapy. Moreover, these hydrogels present great prospects for tissue engineering-based therapies. However, despite many works, there are still some limitations to the use of these materials. Therefore, dual cross-linking is often considered as a method for improving their properties. Furthermore, the complex structure of naturally-derived molecules also favors the simultaneous formation of different non-covalent bonds, e.g., H-bonds often work synergistically with hydrophobic or electrostatic interactions. Moreover, the presence of various interactions and the combination of units sensitive to different stimuli provides a chance to produce multi-responsive hydrogels.

## Figures and Tables

**Table 1:** Comparison of strength and range of the non-covalent interactions.

Interaction	Strength	Description	Example	References
Hydrogen bond	weak (mostly about 20 kJ/mol)	interaction between hydrogen atom (e.g., -OH, -NH_2_) and electronegative atom (e.g., N, O, F)	proteins, nucleic acids, polysaccharides, PVA, PVP, PAAm, UPy	[10,11,19,20,21,22,23,24,25,26,27,28,29,30,31,32,33]
Hydrophobic interactions	medium–strong (stronger than hydrogen bonds and Van der Waals)	interaction between nonpolar moieties of amphiphilic molecules	proteins, PNIPAAm, PEO-PPO-PEO (Pluronic), copolymers PEG-PLA, PEG-PLGA, PEG-DFA, CMC-NIPAAm	[11,34,35,36,37,38,39,40,41,42]
Ionic interactions	relatively strong	based on electrostatic attraction of oppositely charged ions or dipoles	sodium alginate and Ca^2+^; chitosan and phosphate salts/carboxylate salts/polysaccharides; sodium alginate and chitosan	[1,36,43,44,45,46,47,48,49]
Metal–ligand coordination	strong (comparable to the strength of a covalent bond)	interaction between central metal atom or ion and electron donor group(s) (ligands)	ferric ions and catechol ligands; zinc ions and histidine ligands; calcium ions and bisphophonates	[50,51,52,53,54,55,56,57]
Host–guest interaction	wide range of strength	complex hydrogen bonds, Van der Waals, hydrophobic, electrostatic interactions, coordination bonds	cyclodextrins, cucrbiturils, crown ethers, calixarenes, pillarenes	[11,13,36,58,59,60,61,62,63,64,65]

**Table 2:** Recent research on stimuli-sensitive supramolecular hydrogel systems in cancer treatment.

Gelation Trigger	Hydrogel	Therapeutic Agent/Drug	Cell Line (In Vitro)	Cancer (In Vivo)	References
Temperature	HA/PF127	Doxorubicin/ Docetaxel	CT-26	Bowel cancer	[92]
Temperature	GO-FA/HA-CS-g-PNIPAAm	Doxorubicin	MCF-7	Breast cancer	[93]
Temperature	PEG/α-CD	Camptothecin/5-fluorouracil	-	-	[94]
Temperature	PLGA/CS	Paclitaxel	M234-p	Mammary tumor	[95]
pH	α-CD/β-CD/PF127	Doxorubicin	SKOV-3	-	[96]
pH	CS-DA/OP	Doxorubicin	HCT116	-	[97]
pH	CS/PNIPAAm-co-IA	Doxorubicin	MCF-7	Breast cancer	[98]
pH	GC-PF127		H22	Breast cancer	[99]
Temperature-pH	PNIPAAm	Anastrozole	MCF-7	-	[100]
Light	Laponite/α-CD	Doxorubicin Near infrared	HepG2	Liver cancer	[101]
Light	HA/GA/iron ions	Near infrared	KB, 4T1/A375	Breast cancer	[102]
Light	GO/PEG/α-CD	Camptothecin/5-fluorouracil Near infrared	A549	Ascites sarcoma	[103]
Magnetic field	Iron oxide magnetic nanoparticles/CS/DF-PEG-DF	Doxorubicin/ Docetaxel	MDA-MB-231	Breast cancer	[104]
Magnetic field	PEGylated iron oxide nanoparticles/α-CD	Paclitaxel/ Doxorubicin	-	Breast cancer	[105]
Temperature-magnetic field	Magnetic iron oxide nanoparticles/PPZ	Magnetic heat	U87-MG	Glioblastoma	[106]

Abbreviations: HA: hyaluronic acid; PF127: pluronic F127; GO: graphene oxide; FA: folic acid; CS: chitosan; PNIPAAm: poly(*N*-isopropylacrylamide); PEG: poly(ethylene glycol); α-CD: α-cyclodextrin; PLGA: poly(lactide-co-glycolide acid); β-CD: β-cyclodextrin; CS-DA: chitosan-grafted-dihydrocaffeic acid; OP: oxidized pullulan; IA: itaconic acid; GC: glycol chitozan; GA: gallic acid; DF-PEG-DF: telechelic difunctional poly(ethylene glycol); PPZ: poly(organophosphazene).

**Table 3:** Cyclodextrin-based supramolecular hydrogels for gene therapy.

Hydrogel	Vector	Drug	In Vitro	In Vivo	References
PEG-α-CD/CD PPRX	pDNA	-	Colon-26	Male Balb/C mice	[140]
PF68-PLL/α-CD	pDNA	-	mouse fibroblast cells 3T3	-	[141]
MPEG-PCL-PDMAEMA/α-CD	pDNA	-	COS-7	-	[142]
MPEG-PLLD-Arg/α-CD	pMMP-9	-	HNE-1	Nude mice bearing HNE-1 tumors	[143]
MPEG-PCL-PEI-FA/α-CD	pDNA-Nur77	Paclitaxel	HEK293 H460	Male Balb/C nude mice, tumor model	[144]

Abbreviations: PEG: poly(ethylene glycol); α-CD: α-Cyclodextrin; CD: cyclodextrins; PPRX: polypseudorotaxanes; PF68: Pluronic F-68; PLL: poly(L-lysine); MPEG: methoxy-poly(ethylene glycol); PCL: poly(*ε*-caprolactone); PDMAEMA: poly(2-(dimethylamino)ethyl methacrylate); PLLD-Arg: arginine-functionalized poly(L-lysine) dendron; PEI: poly(ethylene imine); FA: folic acid; pDNA: plasmid DNA; Nur 77: Bcl-2 (B-cell lymphoma 2) conversion Nur77 gene; pMMP-9: MMP-9 shRNA plasmid (MMP—matrix metallopeptidase; shRNA: short-hairpin RNA); HNE-1: human nasopharyngeal carcinoma.

## Data Availability

No new data were created or analyzed in this study. Data sharing is not applicable to this article.
